# Self-Perceived Quality of Life and Physical Activity Levels Through Accelerometry in Young People with Intellectual Disabilities

**DOI:** 10.3390/healthcare14060733

**Published:** 2026-03-13

**Authors:** María Menchén-Rubio, Diana Ruiz-Vicente, Ester Jiménez-Ormeño, Teresa García-Pastor

**Affiliations:** 1 Quality of Life of People with Disabilities: Assessment and Intervention (DCVPD), Faculty of Health Sciences-HM Hospitals, University Camilo José Cela, 28692 Madrid, Spain; diana.ruiz@upm.es (D.R.-V.); mariateresa.garcia@uam.es (T.G.-P.); 2 HM Hospitals Health Research Institute, 28050 Madrid, Spain; 3 Department of Physical Education, Sport and Human Movement, Autonomous University of Madrid, 28049 Madrid, Spain; ester.jimenez@uam.es; 4 Faculty of Physical Activity and Sports Sciences (INEF), Technical University of Madrid, 28040 Madrid, Spain; 5 Strength Training and Neuromuscular Performance Research Group (STreNgthP_RG), Faculty of Health Sciences-HM Hospitals, University Camilo José Cela, 28692 Madrid, Spain

**Keywords:** health, accelerometer, WHO-QOL, mild intellectual disability, MVPA, autism spectrum disorder

## Abstract

**Highlights:**

**What are the main findings?**

- Young adults with intellectual disability showed significantly lower self-perceived quality of life—particularly in the social and environmental domains—and lower levels of light physical activity compared to their peers with no disability.
- No associations were found between accelerometer-measured physical activity and quality of life in the group with intellectual disability, whereas vigorous physical activity showed a positive association with the physical QoL domain in the group with no disability.

**What are the implications of the main findings?**

- The absence of PA–QoL associations in young adults with intellectual disability suggests that the benefits of physical activity in this population may depend more on the quality, context, or social meaning of activities rather than on total activity volume.
- Targeted, inclusive physical activity programmes—supported by objective monitoring and adapted to individual needs—may help enhance social participation, environmental satisfaction, and overall quality of life in young adults with intellectual disability.

**Abstract:**

**Background**: The relationship of objectively measured levels of physical activity (PA) to quality of life (QoL) in young adults with intellectual disabilities (IDs) needs to be further researched. This study compares PA levels and self-perceived QoL in young adults with ID compared to those with no intellectual disability and examines whether higher levels of PA are related to better self-perceived QoL in the domains of physical, psychological, social and environmental well-being. **Methods**: A hundred young adults participated (GID: n = 50; GNID: n = 50). Demographic data were collected through questionnaires, and PA levels were measured using ActiGraph GT3X-BT accelerometers over a 7-day period. QoL was assessed using the World Health Organization Quality of Life short questionnaire (WHOQOL-BREF). An independent samples *t*-test was used to examine differences between groups (GID and GNID), and correlations between PA variables and QoL variables were calculated intra-group. The statistical significance was set at *p* ≤ 0.05. **Results**: The GID scored significantly lower in social (*p* = 0.001, d = 0.67), environmental (*p* = 0.007, d = 0.56) and total QoL (*p* = 0.015, d = 0.51) domains, and showed lower light PA (*p* = 0.042, d = 0.45). No significant PA–QoL correlations were found in the GID, while vigorous PA correlated positively with physical QoL in the GNID (rho = 0.35; *p* = 0.028). **Conclusions**: Self-perceived QoL values, as well as PA levels, are lower in young people with ID, with significant differences observed in the social and environmental domains, and in light PA. No associations were found between PA and QoL variables in the group of young people with IDs. Vigorous PA was significantly associated with the physical domain of QoL in the GNID.

## 1. Introduction

Intellectual disability (ID) is manifested through maladaptive behaviour that originates before age 22, stemming from deficits in cognitive, functional and social performance, as well as interactions with their familial, cultural, and institutional environment. Individuals with ID exhibit diminished capacity for reasoning and comprehending information, impeding their academic development and their adaptation and transition to adulthood [1,2]. According to the American Psychiatric Association [1], different degrees of intellectual impairment can be identified, ranging from mild to moderate, severe or profound intellectual disability and a further category known as borderline intellectual functioning.

ID is frequently related to nervous, sensory, metabolic, and physical impairment, which affects the individual’s capacity to function independently [2,3]. Although life expectancy has increased in recent decades, people with ID have a mortality rate between two to four times higher than those who do not, primarily due to cardiovascular diseases [4]. Martínez-Leal et al. [5] reported that the population with ID showed a higher prevalence of obesity, sedentary lifestyles and other conditions such as epilepsy, hypertension, hyperlipidemia and osteoporosis, making health inequalities evident in comparison to those with no disabilities [6,7].

The benefits of physical activity (PA) have been widely demonstrated. The World Health Organization (WHO) provides guidelines for PA in the general population at different ages and, for the first time, for individuals with disabilities [8]. Increased PA has been shown to improve health outcomes in this population group, yet there is scientific evidence that sedentary behaviour and insufficient PA in individuals with ID compared to those with no ID, measured both objectively and subjectively, contribute to the poor health of this population [9,10,11,12,13,14]. In a systematic review by Vancampfort et al. [15], a correlation between more severe ID and poor mobility with lower levels of PA was found, although no results were provided on age categories for adults. The systematic review on PA levels in adults with ID by Dairo et al. [4] concluded that younger adults with mild to moderate ID had significantly higher moderate-vigorous PA than older adults with ID, yet no differences in PA levels compared to similar adults with no ID were found [16,17]. All these studies used objective tools to quantify PA, such as accelerometers, pedometers or both. Significant differences in PA levels were found between adults with ID and the general population when PA questionnaires or diaries were used [18,19].

Quality of life (QoL) is the subjective perception that each individual holds on to their physical, mental, and social well-being. It includes both objective and subjective multidimensional components influenced by personal and environmental factors [20,21,22,23]. Assessment of the various domains of QoL can serve as a tool for identifying the needs of people with disabilities [24]. The World Health Organization Quality of Life questionnaire and its Intellectual Disability Module (WHOQOL-BREF) for individuals with ID is one of the most widely used tools to describe the QoL of people with ID and their families or caregivers [25,26,27].

PA is a significant predictor of improved QoL in people with ID [28,29,30] and ID with autism spectrum disorder (ASD) [31]. A stronger relationship between the total score of QoL and PA levels, when PA is measured using objective methods, has been observed [32,33], although most studies investigating the relationship between PA and QoL do not focus on young adults.

Young adults with ID report lower scores on domains such as emotional and psychological well-being, physical well-being, and environmental quality, using the WHOQOL-BREF [28,31], as well as a significant relationship between QoL scores and self-reports of PA using subjective scales. A study that has evaluated the relationship between objective PA levels and QoL questionnaire (QOL-Q) in participants with ID and ASD used an activity-recording wristband to count steps. It concluded that there was a significant association between total QoL score and weekly step count. However, this objective tool cannot examine different levels of PA intensity [34].

Since scientific literature suggests the need for studies that analyze the relationship between PA and QoL in young adults with ID through an objective assessment of PA levels, the main aims of this study were to compare the levels of PA and perceived QoL in young adults with ID to a similar population with no disability and to examine whether there was a relationship between higher levels of PA and a better self-perception of QoL in its domains of physical health, psychological well-being, social relationships, and environmental quality. The secondary objective was the analyses of the relationships between PA and QoL variables.

## 2. Materials and Methods

### 2.1. Participants

This cross-sectional study recruited 137 potentially eligible participants aged between 18 and 29. The final group of 100 participants was equally divided into two groups: a group with intellectual disability (GID; n = 50) and a group with no intellectual disability (GNID; n = 50). The study sample was obtained through a consecutive sampling method, a non-probabilistic approach, ensuring that every subject was selected in an ordered manner. The sample with no disability was matched for age and gender, as far as possible, to minimize potential confounding variables and enhance the validity of our findings.

Inclusion criteria for both groups were as follows: no health conditions (e.g., asthma, cancer, and heart disease) that could limit PA participation, no physical or sensory impairment (e.g., visual impairment or reduced mobility that prevents walking), and no affiliation to a professional federated sport. The GID inclusion criteria required a documented diagnosis for ID (borderline intelligence, mild, moderate, severe, profound and/or ASD). Sample recruitment took place in the Madrid region (Spain), where GID members were initially contacted by email, informed of the study through meetings in organizations working with ID, as well as educational institutions and universities offering specialized programmes for the ID population. Information on the study was posted on the educational app of the University Camilo José Cela (Madrid, Spain) and members of the GNID group were recruited through said app.

**Figure 1:**
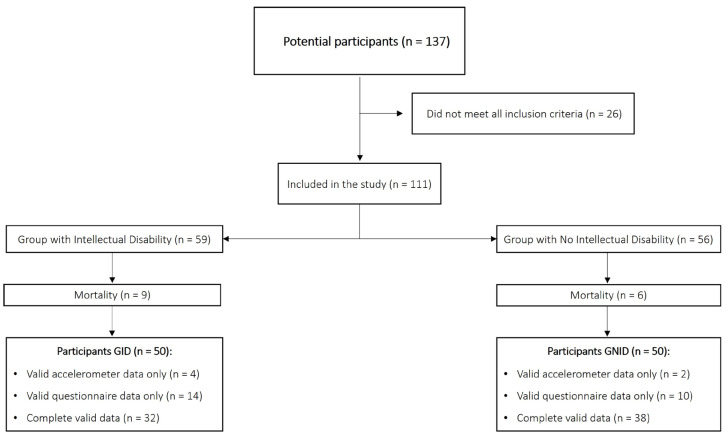
Flow-chart of study participants. GID: group with intellectual disability; GNID: group with no intellectual disability.

Thirty-two GID participants completed the recording of PA levels using accelerometry and the QoL questionnaire. Fourteen participants completed the QoL questionnaire but did not record PA levels, since the data collected did not meet the minimum requirements explained in Section 2.2 (Procedure). Similarly, four participants recorded PA levels but did complete the questionnaire despite having received it. Thirty-eight GNID participants completed the recording of PA levels with accelerometry and the QoL questionnaire. Ten participants completed the QoL questionnaire but did not record PA levels. Two participants recorded PA levels but did not complete the questionnaire. The sample mortality is attributable to participants who enrolled in the study but did not meet all inclusion criteria, or who met the criteria but provided no data for the study variables (Figure 1).

In a previous visit, the participants were informed about the nature of the study. The consent form was signed by the participants themselves, or parents or tutors of participants who have been legally incapacitated. An adapted assent/consent form in simple language with clear graphics was used for GID participants. The study was approved by the University Camilo José Cela Review Board, with approval code 26_22_CAVIDI and carried out in accordance with the latest version of the Declaration of Helsinki.

### 2.2. Procedure

Firstly, the participants completed an online questionnaire based on demographic data: age, gender, type and degree of intellectual disability, residence (family, institutionalized or independent) and parents’ level of education.

QoL was assessed using the Spanish version of the WHOQOL-BREF short questionnaire, created to assess adults with and with no intellectual disabilities [35]. This questionnaire contains a total of 24 questions on four different domains (physical, psychological, social relationships and environment), plus two questions related to general quality of life and satisfaction with health. Each item is based on a 5-point Likert scale ranging from 0 (never) to 4 (almost always), which was reverse-scored and linearly transformed to a 0–100 scale (0 = 100, 1 = 75, 2 = 50, 3 = 25, and 4 = 0); the higher the score, the greater the participants’ QoL (WHOQOL Group, 1998) [35]. This questionnaire was completed by the participants themselves. For the GID, one of the researchers or family members was present to answer any of the questions, even though the questionnaire has been previously used, successfully, with children and young people with ID [36].

Participant PA levels were measured using Actigraph GT3X-BT accelerometers (Actigraph LLC, Pensacola, FL, USA), placed on the hip, for 7 days. It was removed for water sports, sleeping and showering. The measurements were taken over 5 workdays (Monday to Friday) and at the weekend (Saturday and Sunday). Data recording was in periods of 60 s, with a minimum of 10 h/day and at least two full days of use considered to be a valid day [37,38]. The cut-off points applied to classify the intensity of recorded PA were those established by Freedson et al. [39], whose study is a habitual reference for analyzing PA levels in adults with no disabilities [40]. It has also been applied to adults with cardiovascular risk or ASD [41,42,43]. Accelerometers measured sedentary time, light PA, moderate PA, vigorous PA, moderate-vigorous PA (MVPA), and step counts.

### 2.3. Statistical Analysis

A priori sample size estimation for the 2-independent *t*-test using G*Power software (version 3.1, Heinrich Heine University, Düsseldorf, Germany) showed a sample size of 51 would be sufficient when considering a medium effect (g effect size = 0.5) with a one-tailed alpha of 0.05 and a statistical power of 0.8.

Statistical analyses were carried out using the Jamovi statistical software package (version 2.3.21., Sydney, Australia). Significance level was set at *p* ≤ 0.05. Data are presented as mean ± standard deviation (SD) unless stated otherwise. The descriptive variables (age, sex, disability degree and intellectual disability level) were analyzed for groups GID and GNID.

Normality was tested for all variables using the Kolmogorov–Smirnov test. The Levene test was used to verify variable homogeneity. An independent sample *t*-test was used to examine the differences between groups (GID and GNID) in parametric variables related to PA levels and QoL. Welch’s *t*-test was used for parametric variables that did not meet homogeneity (QoL’s total score and environment). For non-parametric variables (sedentary time and vigorous PA), the Mann–Whitney U test was used.

The correlations among PA variables and QoL variables were calculated intra-group, using Pearson’s or Spearman’s rank correlation for parametric and non-parametric variables, respectively. The correlation coefficients were interpreted based on the recommendations of Schober & Schwarte [44], where <0.10 represents negligible correlation, 0.10–0.39 weak correlation, 0.40–0.69 moderate correlation, 0.70 to 0.89 strong correlation, and >0.9 very strong correlation.

## 3. Results

### 3.1. Demographics and Descriptive Data

The study finally included 100 participants aged between 18 and 29. The characteristics of both groups are presented in Table 1.

The descriptive statistics of PA and QoL in both groups are shown in Table 2. There were no significant differences by gender in either group in the reviewed variables (*p* > 0.05).

There were significant differences between groups in social relationships domain (*p* = 0.001), environment (*p* = 0.007), and total score (*p* = 0.015) in QoL, with a medium effect size in the three domains (d = 0.67, d = 0.56, d = 0.51, respectively), showing lower results in the GID. A significantly higher level of light PA was found in the GNID (*p* = 0.042), with a medium effect size (d = 0.45), and, in general, PA levels were higher for the GNID in all variables.

### 3.2. Correlations Between PA Level Variables and QoL Domains

In the GID, several significant positive correlations were found among PA level variables (Table 3). PA moderate average correlated very strongly with MVPA average (r = 0.99; *p* < 0.001) and step count average (r = 0.89; *p* < 0.001), while MVPA average correlated strongly with step count average (r = 0.88; *p* < 0.001). Significant correlations were found between the different domains of the QoL questionnaire. The physical domain correlated strongly with the environmental domain (r = 0.74; *p* < 0.001) and total score (r = 0.88; *p* < 0.001). The psychological domain correlated strongly with total score (r = 0.83; *p* < 0.001). Social relationships correlated strongly with the environmental domain (r = 0.82; *p* < 0.001) and with total score (r = 0.84; *p* < 0.001). The environmental domain correlated very strongly with total score (r = 0.93; *p* < 0.001). No significant correlations were found between QoL variables and PA level variables.

In the GNID, significant correlations were found between PA level variables (Table 4): PA moderate average correlated very strongly with MVPA average (rho = 0.95; *p* < 0.001) and step count average (rho = 0.90; *p* < 0.001). MVPA average correlated strongly with step count average (rho = 0.89; *p* < 0.001). Significant correlations were found between the different domains of the questionnaire regarding QoL. The physical domain correlated strongly with total score (r = 0.79; *p* < 0.001). The psychological domain correlated strongly with the environmental domain (r = 0.73; *p* < 0.001) and total score (r = 0.91; *p* < 0.001). Social relationships also correlated strongly with the environmental domain and very strongly with total score (r = 0.80; *p* < 0.001). A positive correlation was found between the QoL variables and PA level. There was a weak correlation between vigorous PA and the physical domain of the QoL questionnaire (rho = 0.35; *p* = 0.028).

## 4. Discussion

The main aims of this study were to compare the levels of PA and self-perceived QoL in young adults with ID to a similar population with no intellectual disability and to examine whether there was any relationship between higher levels of PA and a better self-perception of QoL in different domains. Our study demonstrates that young adults with ID report lower PA levels and perceived QoL compared to young adults with no disability, highlighting a gap in adherence to WHO recommendations for physical activity levels in young adults. Correlations between PA variables and QoL variables were found in both groups, yet no correlations were found between the two, except in the GNID, where a significant correlation was observed between vigorous PA and the physical domain of QoL.

Accelerometer data showed that the GID had significantly lower light PA (226.50 vs. 188.15 min/day, respectively) compared to young adults with no ID. Although the remaining PA variables also report lower GID means, no other significant differences between groups were found. These results are consistent with Frey [16], whose study on adults with and with no ID reported no differences in PA levels measured with accelerometers between groups. The MVPA levels of the GID (44.67 ± 18.11 min/day) are higher than those reported by Barnes et al. [45] (176.7 min/week), Dixon-Ibarra et al. [46] (21 min/day), Oviedo et al. [47] (36.3 min/day) and Hsu et al. [38] (16.67 min/day). The samples in these studies were of a higher mean age, except in Barnes et al. [45], where the mean age matches our sample. Most of the participants in these studies had moderate ID, whereas in our study only five participants had moderate ID. According to Dairo et al. [4], younger adults with mild to moderate ID reported significantly higher moderate-vigorous PA compared to older adults with ID. Thus, the severity of ID is a good predictor for minimum levels of recommended PA. Most of our participants had mild levels of ID, which may explain the lack of significant differences in MVPA variables compared to the GNID, although our findings state that they are less active than the general population. On the other hand, Leung et al. [48], in their systematic review, state that the diversity of findings in PA levels of adults with ID may be attributed to the variation in protocols used with accelerometers, and the different models used. Only one of the articles in their review focused exclusively on young adults, a case study that used a different accelerometer model from the one used in our study [49], making comparisons with our results challenging.

Other studies have concluded that individuals with ID and those with ASD have lower scores in several QoL domains [28,31,34]. These findings are consistent with our results in the domains of social relationships, environment, and the total score. Our findings are also consistent with the study conducted by Biggs & Carter [24] on young people with ID and ASD transitioning to adulthood, which showed that the lowest scores were in the social domain and were significantly different compared to the sample with no disability. Their study differs in the physical and psychological domains, where they also reported significantly lower results. This discrepancy with our results may be due to the fact that their questionnaire was completed by parents or caregivers, whereas individuals with ID tend to report greater self-perception of their QoL than their caregivers [50,51,52], which may have influenced the non-existent correlation with PA levels in the GID, since the answers to our QoL questionnaire were provided by the participants themselves. This contrasts with the findings in the GNID, where correlations were found between vigorous PA and the physical domain of the QoL questionnaire.

Hamm & Yun [31] also found associations between PA and all domains of QoL in young people with and with no ASD, whereas Anokye et al. [32] demonstrated that only higher levels of PA were associated with a better self-perception of QoL in neurotypical adults. However, these authors used different accelerometer models and a different QoL questionnaire. The older age of their sample may also account for the differences with our results, which do not align with our conclusions. Our study answers the recommendation to study the relationship between PA and QoL across different age ranges in adulthood [34] and provides insights into young adults with ID, where PA does not show a correlation with QoL levels when using objective measures of PA. The lack of association between PA levels and QoL in the ID group suggests that the ‘quantity’ of activity may be secondary to its ‘quality’ or social significance. However, we must be cautious since the non-significant correlations in this study do not necessarily indicate the absence of a true relationship due to the small effective sample size and the self-assessment limitations mentioned above. Longitudinal data indicate that, overall, PA levels do not always translate into proportional health-related fitness improvements in adolescents with disabilities, reinforcing the critical importance of intervention specificity [53]. Several studies on university students with no intellectual disability have found that higher levels of PA, measured with both questionnaires and accelerometers, were linked to a better perception of QoL, especially in the physical, social, and psychological domains [27,54,55]. This is consistent with our GNID findings, which show a positive correlation between vigorous PA and the physical domain of QoL.

The strengths of our study are: first, the use of accelerometers to objectively determine levels of PA in young adults, along with a validated QoL questionnaire proposed by the WHO; second, PA and QoL variables were examined within a stipulated age group avoiding the typical biases often linked to age heterogeneity. A matched group with no disability was included for comparison with the general population.

There are limitations in our study that should be discussed. The sample sizes for associations were relatively small in both groups, as not all participants completed requirements for data inclusion (questionnaire and accelerometer), meaning the current findings should be generalized with caution; however, the effect of the bias was similar in both groups, protecting the internal validity of the study. Since our study is cross-sectional, causal relationships could not be established, nor could we determine whether PA levels, which impact directly on QoL, were habitual or incidental. Although a high percentage of the population with ID identifies as having mild ID, like our GID, only a small percentage of this population accesses university programmes. Our GID may not be representative of the entire population with ID due to their sociodemographic characteristics. Matey-Rodríguez et al. [56] reported higher cut-off points in PA measurement with ActiGraph triaxial accelerometer models compared to those proposed by Freedson et al. [39], who used older uniaxial, omnidirectional, or biaxial models in the general population. We were also unable to follow the recommendation by Santos-Lozano et al. [57] to use specific cut-off triaxial accelerometer points in young adults with ID, since cut-off points for this population do not exist. This limitation aligns with McGarty et al. [58], who emphasized that the cut-off points commonly applied to the general population may not be appropriate for individuals with ID, particularly for moderate and vigorous PA levels, highlighting the need for ID-specific cut-off points across different age groups. This could explain why no significant differences in PA levels were found between groups, as a single classification was used for two different population groups.

The lower PA levels in youth with ID and the positive association observed between vigorous PA and the physical domain of QoL in young adults with no ID, targeted strategies to increase engagement in higher-intensity activities would be beneficial for young adults with ID. Moreover, the lower PA levels and QoL scores observed in youth with ID necessitate a multidimensional public health response. Therefore, it would be advisable to promote the design of inclusive PA and sport qualitymes, supported by objective monitoring tools such as accelerometry, aimed at improving physical, cognitive and emotional components within socially enriching environments, ensuring that physical activity serves as a bridge to health equity and life satisfaction for people with disabilities [53]. In addition, these programmes should consider the needs and abilities of young people with ID to increase the social and psychological domains of QoL [59]. Family members and caregivers should also be involved in these initiatives to address discrepancies in QoL perceptions. Increasing PA levels in this population would not only improve their physical and psychological well-being but also contribute to greater social inclusion and overall life satisfaction. Research on age and appropriate PA cut-off points could enhance the accuracy of activity assessment and contribute to intervention design.

## 5. Conclusions

In conclusion, the GID exhibited poorer outcomes across all domains of self-perceived QoL, with significant differences observed in the social and environmental domains, as well as in the total QoL score. They also exhibited lower PA levels, although the difference was significant only for light PA. No association was found between PA levels and QoL in the GID. A weak association between vigorous PA and the physical domain of the QoL questionnaire was observed in the GNID.

## Figures and Tables

**Table 1:** Characteristics of participants (n = 100).

	GID		GNID	
	n = 50	Missing (n)	n = 50	Missing (n)
**Age (years)**	21.7 ± 3.09	2	20.9 ± 3.03	2
**Sex**				
Male	30	-	23	-
Female	20	-	27	-
**Disability degree (%)**	55.6 ± 14.6	9	-	-
**ID level**		6		
Borderline Intellectual Functioning	10	-	-	-
Mild	21	-	-	-
Moderate	5	-	-	-
Autism Spectrum Disorder	8	-	-	-
**Residence**		7		7
Family	40	-	25	-
Institutionalized	3	-	-	-
Independent	-	-	18	-
**Parental education level**		11		10
<College/University	20	-	14	-
≥College/University	19	-	26	-

ID: intellectual disability; GID: group with intellectual disability; GNID: group with no intellectual disability.

**Table 2:** Physical activity level and quality of life in young adults with and with no intellectual disability.

Variables	GID	GNID	*p*	d
**PA Level**	n = 37	n = 46		
Sedentary time (h/day)	9.77 ± 3.67	9.29 ± 1.66	0.810	0.04
Step counts (steps/day)	7952.12 ± 2530.75	8305.47 ± 2647.31	0.550	0.12
Light PA (min/day)	188.15 ± 96.55	226.50 ± 71.21	**0.042**	0.45
Moderate PA (min/day)	42.70 ± 17.35	45.41 ± 21.11	0.541	0.12
Vigorous PA (min/day)	1.67 ± 1.99	2.87 ± 4.91	0.601	0.07
MVPA (min/day)	44.67 ± 18.11	48.74 ± 22.68	0.388	0.18
**Self-report QoL (range 0–100)**	n = 47	n = 49		
Physical	66.79 ± 17.09	69.10 ± 14.68	0.480	0.28
Psychological	62.16 ± 19.68	69.06 ± 17.37	0.071	0.40
Social relationships	59.22 ± 24.89	74.24 ± 19.87	**0.001**	0.67
Environment	61.17 ± 22.65	71.80 ± 13.97	**0.007**	0.56
Total Score	90.60 ± 18.32	98.47 ± 11.99	**0.015**	0.51

GID: group with intellectual disability; GNID: group with no intellectual disability; PA: physical activity; QoL: quality of life. Bold text indicates statistical significance in the comparison.

**Table 3:** The correlations among physical activity (PA) and quality of life (QoL) in young adults with intellectual disability.

Variables	(1)	(2)	(3)	(4)	(5)	(6)	(7)	(8)	(9)	(10)
**Physical Activity Level (PA; counts)**										
(1) Sedentary time	-	-	-	-	-	-	-	-	-	-
(2) Step counts	−0.37 *	-	-	-	-	-	-	-	-	-
(3) Light PA	−0.40 *	0.67 ***	-	-	-	-	-	-	-	-
(4) Moderate PA	−0.23	0.88 ***	0.42 *	-	-	-	-	-	-	-
(5) Vigorous PA	−0.01	0.35 *	0.05	0.40 *	-	-	-	-	-	-
(6) MVPA	−0.23	0.87 ***	0.39 *	0.98 ***	0.47 ***	-	-	-	-	-
**Self-report quality of life (QoL; range 0–100)**										
(7) Physical	−0.11	−0.01	−0.24	0.12	0.32	0.13	-	-	-	-
(8) Psychological	−0.12	−0.14	−0.08	−0.12	0.14	−0.13	0.63 ***	-	-	-
(9) Social relationships	−0.01	−0.03	−0.23	0.03	0.29	0.03	0.71 ***	0.63 ***	-	-
(10) Environment	−0.04	−0.01	−0.24	0.07	0.25	0.07	0.77 ***	0.65 ***	0.81 ***	-
(11) Total score	−0.09	−0.03	−0.23	0.05	0.28	0.05	0.89 ***	0.83 ***	0.85 ***	0.92 ***

PA: physical activity; QoL, quality of life; MVPA, moderate-vigorous physical activity; * *p* < 0.05; *** *p* < 0.001.

**Table 4:** The correlations among physical activity (PA) and quality of life (QoL) in young adults with no intellectual disability.

Variables	(1)	(2)	(3)	(4)	(5)	(6)	(7)	(8)	(9)	(10)
**Physical Activity Level (PA; counts)**										
(1) Sedentary time	-	-	-	-	-	-	-	-	-	-
(2) Step counts	−0.38 *	-	-	-	-	-	-	-	-	-
(3) Light PA	−0.22	0.36 *	-	-	-	-	-	-	-	-
(4) Moderate PA	−0.50 **	0.91 ***	0.13	-	-	-	-	-	-	-
(5) Vigorous PA	0.05	0.21	0.34 *	0.19	-	-	-	-	-	-
(6) MVPA	−0.43 **	0.89 ***	0.17	0.95 ***	0.38 *	-	-	-	-	-
**Self-report quality of life (QoL; range 0–100)**										
(7) Physical	−0.14	0.08	−0.23	0.07	0.35 *	0.19	-	-	-	-
(8) Psychological	0.10	0.06	−0.03	0.04	0.26	0.14	0.58 ***	-	-	-
(9) Social relationships	0.14	0.10	−0.13	−0.02	0.12	0.07	0.31	0.56 ***	-	-
(10) Environment	0.21	−0.11	−0.16	−0.15	0.11	−0.04	0.39 *	0.71 ***	0.46 **	-
(11) Total score	0.09	0.02	−0.14	−0.02	0.27	0.09	0.78 ***	0.90 ***	0.61 ***	0.78 ***

PA: physical activity; QoL, quality of life; * *p* < 0.05; ** *p* < 0.01; *** *p* < 0.001.

## Data Availability

The data that support the findings of this study are available from the corresponding author upon reasonable request.

## Abbreviations

The following abbreviations are used in this manuscript:

ASD	Autism spectrum disorder
GID	Group with intellectual disability
GNID	Group with no intellectual disability
ID	Intellectual disability
MVPA	Moderate-vigorous physical activity
PA	Physical activity
QoL	Quality of life
WHO	World Health Organization
WHOQOL-BREF	World Health Organization Quality of Life short questionnaire

## References

[1] American Psychiatric Association (2013). Diagnostic and Statistical Manual of Mental Disorders.

[2] Schalock R.L., Luckasson R., Tassé M.J. (2021). An Overview of Intellectual Disability: Definition, Diagnosis, Classification, and Systems of Supports (12th Ed.). Am. J. Intellect. Dev. Disabil..

[3] Luckasson R., Schalock R.L. (2013). Defining and Applying a Functionality Approach to Intellectual Disability. J. Intellect. Disabil. Res..

[4] Dairo Y.M., Collett J., Dawes H., Oskrochi G.R. (2016). Physical Activity Levels in Adults with Intellectual Disabilities: A Systematic Review. Prev. Med. Rep..

[5] Martínez-Leal R., Salvador-Carulla L., Linehan C., Walsh P., Weber G., Van Hove G., Määttä T., Azema B., Haveman M., Buono S. (2011). The Impact of Living Arrangements and Deinstitutionalisation in the Health Status of Persons with Intellectual Disability in Europe. J. Intellect. Disabil. Res..

[6] Harris J.C., Greenspan S., Singh N.N. (2016). Definition and Nature of Intellectual Disability. Handbook of Evidence-Based Practices in Intellectual and Developmental Disabilities.

[7] Jacinto M., Matos R., Gomes B., Caseiro A., Antunes R., Monteiro D., Ferreira J.P., Campos M.J. (2023). Physical Fitness Variables, General Health, Dementia and Quality of Life in Individuals with Intellectual and Developmental Disabilities: A Cross-Sectional Study. Healthcare.

[8] Bull F.C., Al-Ansari S.S., Biddle S., Borodulin K., Buman M.P., Cardon G., Carty C., Chaput J.-P., Chastin S., Chou R. (2020). World Health Organization 2020 Guidelines on Physical Activity and Sedentary Behaviour. Br. J. Sports Med..

[9] Draheim C.C., Williams D.P., McCubbin J.A. (2002). Prevalence of Physical Inactivity and Recommended Physical Activity in Community-Based Adults with Mental Retardation. Ment. Retard..

[10] Finlayson J., Jackson A., Cooper S.A., Morrison J., Melville C., Smiley E., Allan L., Mantry D. (2009). Understanding Predictors of Low Physical Activity in Adults with Intellectual Disabilities. J. Appl. Res. Intellect. Disabil..

[11] Robertson J., Emerson E., Gregory N., Hatton C., Turner S., Kessissoglou S., Hallam A. (2000). Lifestyle Related Risk Factors for Poor Health in Residential Settings for People with Intellectual Disabilities. Res. Dev. Disabil..

[12] Zwack C.C., McDonald R., Tursunalieva A., Lambert G.W., Lambert E.A. (2022). Exploration of Diet, Physical Activity, Health Knowledge and the Cardiometabolic Profile of Young Adults with Intellectual Disability. J. Intellect. Disabil. Res..

[13] Diaz K.M. (2020). Leisure-Time Physical Activity and All-Cause Mortality among Adults with Intellectual Disability: The National Health Interview Survey. J. Intellect. Disabil. Res..

[14] Jacob U.S., Pillay J., Johnson E., Omoya O., Adedokun A.P. (2023). A Systematic Review of Physical Activity: Benefits and Needs for Maintenance of Quality of Life among Adults with Intellectual Disability. Front. Sports Act. Living.

[15] Vancampfort D., Van Damme T., Firth J., Stubbs B., Schuch F., Suetani S., Arkesteyn A., Van Biesen D. (2022). Physical Activity Correlates in Children and Adolescents, Adults, and Older Adults with an Intellectual Disability: A Systematic Review. Disabil. Rehabil..

[16] Frey G.C. (2004). Comparison of Physical Activity Levels between Adults with and without Mental Retardation. J. Phys. Act. Health.

[17] Stanish H.I. (2004). Accuracy of Pedometers and Walking Activity in Adults with Mental Retardation. Adapt. Phys. Act. Q..

[18] Hawkins A., Look R. (2006). Levels of Engagement and Barriers to Physical Activity in a Population of Adults with Learning Disabilities. Br. J. Learn. Disabil..

[19] Emerson E. (2005). Underweight, Obesity and Exercise among Adults with Intellectual Disabilities in Supported Accommodation in Northern England. J. Intellect. Disabil. Res..

[20] Schalock R.L., Verdugo M.Á. (2007). El Concepto de Calidad de Vida En Los Servicios y Apoyos Para Personas Con Discapacidad Intelectual.

[21] Amor A.M., Verdugo M., Fernández M., Aza A., Sánchez-Gómez V., Wolowiec Z. (2023). Development and Validation of Standardized Quality of Life Measures for Persons with IDD. Behav. Sci..

[22] Schalock R.L., Angel M., Alonso V., Braddock D.L. (2002). Handbook on Quality of Life for Human Service Practitioners.

[23] Chen T.H., Li L., Kochen M.M. (2005). A Systematic Review: How to Choose Appropriate Health-Related Quality of Life (HRQOL) Measures in Routine General Practice?. J. Zhejiang Univ. Sci. B.

[24] Biggs E.E., Carter E.W. (2016). Quality of Life for Transition-Age Youth with Autism or Intellectual Disability. J. Autism Dev. Disord..

[25] Özer D., Süngü B., Aydoğan A., Özer Tekin N.B., Kapucuoğlu T., Namli Şeker A., Şimşek Zeytin D.E., Zeynel Bingöl Z. (2024). The Effects of Online Intervention Program Implemented during COVID-19 on the Quality of Life and Physical Activities of Individuals with Intellectual Disabilities and Their Mothers. J. Intellect. Disabil..

[26] Alnahdi G.H., Alwadei A., Woltran F., Schwab S. (2022). Measuring Family Quality of Life: Scoping Review of the Available Scales and Future Directions. Int. J. Environ. Res. Public Health.

[27] Dana A., Ranjbari S., Mosazadeh H., Maliszewski W.J., Błachnio A. (2022). Correlations of Accelerometer-Measured Physical Activity with Body Image and Quality of Life among Young and Older Adults: A Pilot Study. Int. J. Environ. Res. Public Health.

[28] Carbó-Carreté M., Guàrdia-Olmos J., Giné C., Schalock R.L. (2016). A Structural Equation Model of the Relationship between Physical Activity and Quality of Life. Int. J. Clin. Health Psychol..

[29] Yang W., Yu J.J., Wong S.H.S., Sum R.K.W., Li M.H., Sit C.H.P. (2022). The Associations among Physical Activity, Quality of Life, and Self-Concept in Children and Adolescents with Disabilities: A Moderated Mediation Model. Front. Pediatr..

[30] St. John L., Borschneck G., Cairney J. (2020). A Systematic Review and Meta-Analysis Examining the Effect of Exercise on Individuals with Intellectual Disability. Am. J. Intellect. Dev. Disabil..

[31] Hamm J., Yun J. (2017). Influence of Physical Activity on the Health-Related Quality of Life of Young Adults with and without Autism Spectrum Disorder. Disabil. Rehabil..

[32] Anokye N.K., Trueman P., Green C., Pavey T.G., Taylor R.S. (2012). Physical Activity and Health Related Quality of Life. BMC Public Health.

[33] Marquez D.X., Aguinãga S., Vásquez P.M., Conroy D.E., Erickson K.I., Hillman C., Stillman C.M., Ballard R.M., Sheppard B.B., Petruzzello S.J. (2020). A Systematic Review of Physical Activity and Quality of Life and Well-Being. Transl. Behav. Med..

[34] Tomaszewski B., Savage M.N., Hume K. (2022). Examining Physical Activity and Quality of Life in Adults with Autism Spectrum Disorder and Intellectual Disability. J. Intellect. Disabil..

[35] World Health Organization (1998). WHOQOL User Manual: Programme on Mental Health.

[36] Lin J.D., Hu J., Yen C.F., Hsu S.W., Lin L.P., Loh C.H., Chen M.H., Wu S.R., Chu C.M., Wu J.L. (2009). Quality of Life in Caregivers of Children and Adolescents with Intellectual Disabilities: Use of WHOQOL-BREF Survey. Res. Dev. Disabil..

[37] Zhu X., Haegele J.A., Wang D., Zhang L., Wu X. (2020). Reactivity to Accelerometer Measurement of Youth with Moderate and Severe Intellectual Disabilities. J. Intellect. Disabil. Res..

[38] Hsu P.J., Chou H.S., Pan Y.H., Ju Y.Y., Tsai C.L., Pan C.Y. (2021). Sedentary Time, Physical Activity Levels and Physical Fitness in Adults with Intellectual Disabilities. Int. J. Environ. Res. Public Health.

[39] Freedson P.S., Melanson E., Sirard J. (1998). Calibration of the Computer Science and Applications, Inc. Accelerometer. Med. Sci. Sports Exerc..

[40] Zhou Y., Huang Z., Liu Y., Liu D. (2024). The Effect of Replacing Sedentary Behavior with Different Intensities of Physical Activity on Depression and Anxiety in Chinese University Students: An Isotemporal Substitution Model. BMC Public Health.

[41] Carrera-Bastos P., Rydhög B., Fontes-Villalba M., Arvidsson D., Granfeldt Y., Sundquist K., Jönsson T. (2024). Randomised Controlled Trial of Lifestyle Interventions for Abdominal Obesity in Primary Health Care. Prim. Health Care Res. Dev..

[42] Garcia-Pastor T., Salinero J.J., Theirs C.I., Ruiz-Vicente D. (2019). Obesity Status and Physical Activity Level in Children and Adults with Autism Spectrum Disorders: A Pilot Study. J. Autism Dev. Disord..

[43] Sebastian D.R., Lucía B., Gastón B., Fabian M., Yanina Luciana M., Maria Pilar A., Nilda Raquel P. (2022). Associations between Objectively Measured Physical Activity, Sedentary Time, and Cardiorespiratory Fitness with Inflammatory and Oxidative Stress Markers and Heart Rate Variability. J. Public Health Res..

[44] Schober P., Schwarte L.A. (2018). Correlation Coefficients: Appropriate Use and Interpretation. Anesth. Analg..

[45] Barnes T.L., Howie E.K., McDermott S., Mann J.R. (2013). Physical Activity in a Large Sample of Adults with Intellectual Disabilities. J. Phys. Act. Health.

[46] Dixon-Ibarra A., Lee M., Dugala A. (2013). Physical Activity and Sedentary Behavior in Older Adults with Intellectual Disabilities: A Comparative Study. Adapt. Phys. Activ. Q..

[47] Oviedo G.R., Travier N., Guerra-Balic M. (2017). Sedentary and Physical Activity Patterns in Adults with Intellectual Disability. Int. J. Environ. Res. Public Health.

[48] Leung W., Siebert E.A., Yun J. (2017). Measuring Physical Activity with Accelerometers for Individuals with Intellectual Disability: A Systematic Review. Res. Dev. Disabil..

[49] Lante K.A., Walkley J.W., Gamble M., Vassos M.V. (2011). An Initial Evaluation of a Long-Term, Sustainable, Integrated Community-Based Physical Activity Program for Adults with Intellectual Disability. J. Intellect. Dev. Disabil..

[50] McPhail S., Beller E., Haines T. (2008). Two Perspectives of Proxy Reporting of Health-Related Quality of Life Using the Euroqol-5D, an Investigation of Agreement. Med. Care.

[51] Simeoni M.C., Schmidt S., Muehlan H., Debensason D., Bullinger M., Petersen C., Quittan M., Schuhfried O., Orbicini D., Thyen U. (2007). Field Testing of a European Quality of Life Instrument for Children and Adolescents with Chronic Conditions: The 37-Item DISABKIDS Chronic Generic Module. Qual. Life Res..

[52] Bastiaansen D., Koot H.M., Ferdinand R.F., Verhulst F.C. (2004). Quality of Life in Children with Psychiatric Disorders: Self-, Parent, and Clinician Report. J. Am. Acad. Child Adolesc. Psychiatry.

[53] Suarez-Villadat B., Montero M., Montero S., López-García A., Villagra A. (2025). Adapted Judo as a Multidimensional Intervention: Effects on Physical Fitness and Psychosocial Well-Being in Adolescents with Down Syndrome. Healthcare.

[54] Gill D.L., Hammond C.C., Reifsteck E.J., Jehu C.M., Williams R.A., Adams M.M., Lange E.H., Becofsky K., Rodriguez E., Shang Y.T. (2013). Physical Activity and Quality of Life. J. Prev. Med. Public Health.

[55] Krzepota J., Biernat E., Florkiewicz B. (2015). The Relationship between Levels of Physical Activity and Quality of Life among Students of the University of the Third Age. Cent. Eur. J. Public Health.

[56] Matey-Rodríguez C., López-Ortiz S., Peñín-Grandes S., Pinto-Fraga J., Valenzuela P.L., Pico M., Fiuza-Luces C., Lista S., Lucia A., Santos-Lozano A. (2023). Validation and Determination of Physical Activity Intensity GT3X+ Cut-Points in Children and Adolescents with Physical Disabilities: Preliminary Results in a Cerebral Palsy Population. Children.

[57] Santos-Lozano A., Santín-Medeiros F., Cardon G., Torres-Luque G., Bailón R., Bergmeir C., Ruiz J.R., Lucia A., Garatachea N. (2013). Actigraph GT3X: Validation and Determination of Physical Activity Intensity Cut Points. Int. J. Sports Med..

[58] McGarty A.M., Penpraze V., Melville C.A. (2016). Calibration and Cross-Validation of the ActiGraph WGT3X+ Accelerometer for the Estimation of Physical Activity Intensity in Children with Intellectual Disabilities. PLoS ONE.

[59] Suarez-Villadat B., Villagra A., Veiga O.L., Cabanas-Sanchez V., Izquierdo-Gomez R. (2021). Prospective Associations of Physical Activity and Health-Related Physical Fitness in Adolescents with Down Syndrome: The UP&DOWN Longitudinal Study. Int. J. Environ. Res. Public Health.

